# Fatigue Behavior of Porous Ti-6Al-4V Made by Laser-Engineered Net Shaping

**DOI:** 10.3390/ma11020284

**Published:** 2018-02-12

**Authors:** Seyed Mohammad Javad Razavi, Giancarlo G. Bordonaro, Paolo Ferro, Jan Torgersen, Filippo Berto

**Affiliations:** 1 Department of Mechanical and Industrial Engineering, Norwegian University of Science and Technology (NTNU), 7491 Trondheim, Norway; javad.razavi@ntnu.no (S.M.J.R.); jan.torgersen@ntnu.no (J.T.); 2 Institute for Mechanical Engineering and Materials Technology, University of Applied Sciences of Southern Switzerland, CH-6928 Manno, Switzerland; giancarlo.bordonaro@supsi.ch; 3 Department of Engineering and Management, University of Padova, 36100 Vicenza, Italy; ferro@gest.unipd.it

**Keywords:** fatigue, Direct Energy Deposition (DED), Laser Engineered Net Shaping (LENS), Ti-6Al-4V, additive manufacturing, porosity

## Abstract

The fatigue behavior and fracture mechanisms of additively manufactured Ti-6Al-4V specimens are investigated in this study. Three sets of testing samples were fabricated for the assessment of fatigue life. The first batch of samples was built by using Laser-Engineered Net Shaping (LENS) technology, a Direct Energy Deposition (DED) method. Internal voids and defects were induced in a second batch of samples by changing LENS machine processing parameters. Fatigue performance of these samples is compared to the wrought Ti-6Al-4V samples. The effects of machine-induced porosity are assessed on mechanical properties and results are presented in the form of SN curves for the three sets of samples. Fracture mechanisms are examined by using Scanning Electron Microscopy (SEM) to characterize the morphological characteristics of the failure surface. Different fracture surface morphologies are observed for porous and non-porous specimens due to the combination of head write speed and laser power. Formation of defects such as pores, unmelted regions, and gas entrapments affect the failure mechanisms in porous specimens. Non-porous specimens exhibit fatigue properties comparable with that of the wrought specimens, but porous specimens are found to show a tremendous reduced fatigue strength.

## 1. Introduction

Additive manufacturing (AM) is a rapidly emerging technology for the fabrication of three-dimensional objects. Unlike traditional manufacturing processes based on material subtraction (e.g., milling, turning, cutting) and forming methods (e.g., foundry, molding, stamping), AM processes are capable of building 3D geometries directly from digital models. Laser-Engineered Net Shaping (LENS) is a directed energy deposition method (DED). DED is commonly used for repair operations or to add additional material to existing components [1,2,3,4,5]. A nozzle is mounted on a multi-axis arm, which deposits melted material onto a specified surface, where it solidifies. The nozzle can move in multiple directions, and material can be deposited from any angle spanned by a 4- or 5-axis machine. Commonly recognized benefits of AM technology include the capabilities to produce components with any type of geometrical complexity and without significantly increasing of the actual costs for the parts. Design rules based on constraints of molds and dies are actually eliminated with the great benefit of no additional effort for more complex designs enabling the production of competitive, low-volume, and highly customized parts. Despite these advantages, the adoption of this technology for mass production is limited by the size of the objects that can be made, the costs and size of the machines and materials used, the accuracy and surface finish that can be achieved, and the certification standards that have to be met. Developing standards is a critical challenge that must be addressed to achieve a wide diffusion and adoption of this technology. Standards would provide a certified guideline for creating products that conform to certain specifications and ensure compatibility to quality, safety, and reliability performance. Before AM products can be safely employed in major industrial sectors like aerospace, automotive, or medical, physical properties have to be characterized and fatigue strength has to be investigated with respect to conventionally manufactured materials. At present, the basic understanding of the fatigue behavior of AM materials must be substantially improved at all scale levels before the unique features of this rapidly developing technology can be used in critical load bearing applications. The fatigue design and quality assurance of AM components cannot be performed accurately due to the novelty of this technology and hence the very limited experienced and available empirical database of fatigue behavior of such components comprising high geometrical complexity and unusual, production specific internal structure [6,7,8]. A fundamental understanding of basic fatigue phenomena plays an important role in a safe design and implementation in industrial applications. Standard test methods are currently applied to conventional metallic materials for the measurements of mechanical, fatigue and fracture properties. Similar methods are being developed for AM materials to provide additional details such as anisotropy effects due to the layered deposition methods and the presence of manufacturing defects, inclusions, flaws, cracks, and porosities. An investigation of the fatigue behavior and failure mechanisms of Direct Energy Deposited Ti-6Al-4V specimens via Laser Engineered Net Shaping (LENS) AM process has been conducted by Sterling et al. [9,10]. They fabricated and machined “Dog-bone” fatigue specimens in dimensions in conformance to ASTM standards with two sets of LENS parameters and compared the results with the wrought counterparts. Cyclic stress-strain Ramberg-Osgood curves showed that fatigue lives of the LENS Ti-6Al-4V materials are lower than those of wrought counterparts originating in the LENS specific porosity and microstructure. The fatigue curves generated using the Coffin-Manson approach showed that the behavior of both LENS Ti-6Al-4V series is primarily elastic, with little noticeable plastic strain occurring at 1% strain amplitude. The wrought Ti-6Al-4V experiences more plastic strain than both LENS materials, with noticeable amounts starting at 0.8% strain amplitude. Different microstructures and porosity do affect the fatigue life and failure mechanisms of LENS parts, encouraging further efforts in creating and employing a microstructure-sensitive fatigue model for predicting fatigue life of AM-fabricated parts. Zhai et al. [11] studied the fatigue crack growth (FCG) of LENS and EBM Ti-6Al-4V specimens fabricated with low laser power (LP) and high laser power (HP). LENS HP fabrication yielded higher FCG threshold values in Ti-6Al-4V than LP fabrication. FCG mechanisms were correlated primarily with α/α’ phases and colonies in both LENS samples. Fatigue damage mechanisms were also correlated to the materials’ characteristic microstructural features, and crack interaction with fine α morphologies was reasoned as the primary mechanism in all regions of fatigue crack growth. Prabhu et al. [12] investigated LENS-deposited Ti-6Al-4V for high-cycle fatigue life in as-built and simulated repair conditions. Both fatigue life and crack initiation in the LENS deposits are predominately controlled by the presence of defects like unmelted particles at the surface. Li et al. [13] conducted an examination of published fatigue data of AM alloys of significant technological interest. AM Ti-6Al-4Vs fatigue performance is governed by the same features controlling traditional cast and wrought products, i.e., surface finish, residual stress, internal defects, and microstructure. Fatigue performance of DED manufactured specimens with post processing surface treatment are demonstrated to be superior to wrought and annealed Ti-6Al-4V materials, most likely due to the finer microstructure that can be achieved with AM processing. However, as-built DED parts exhibit considerable roughness which would significantly decrease the fatigue performance. 

In this work, Ti-6Al-4V specimens are fabricated by LENS and the fatigue strength is compared to specimens fabricated by conventional manufacturing. LENS samples were fabricated by selecting two different sets of AM parameters. The first set of parameters was selected as an optimal choice in order to minimize the presence of internal defects. A second batch of samples was fabricated by slightly deviating from the optimal parameters to introduce tailored internal defects such as voids and porosities. Effects of porosity levels induced by parameter variations are linked to fatigue performances and other mechanical properties. A third batch of Ti-6Al-4V specimens was fabricated with traditional manufacturing processes and tested for comparison.

## 2. Materials and Methods

### 2.1. Fabrication Conditions and Preparation

Spherical Ti-6Al-4V Grade 5 powders with a mesh size of −100/+325 (SAE AMS 4998C) provided by TIMET^®^ (Dallas, TX, USA) were used for LENS fabrication of specimens. LENS parts were fabricated using an LENSTM 850-R machine (Optomec^®^, Albuquerque, NM, USA) powered by a 1 kW Nd:YAG laser. The first batch of samples was built with a set of processing parameters to ensure near-zero defects and full density deposited layers. The second batch of samples was built slightly changing processing parameters to induce voids and porosities across each layer of the parts. Table 1 shows the processing parameters used for the fabrication of the two batches of samples by LENS technology. 

**Figure 1:**
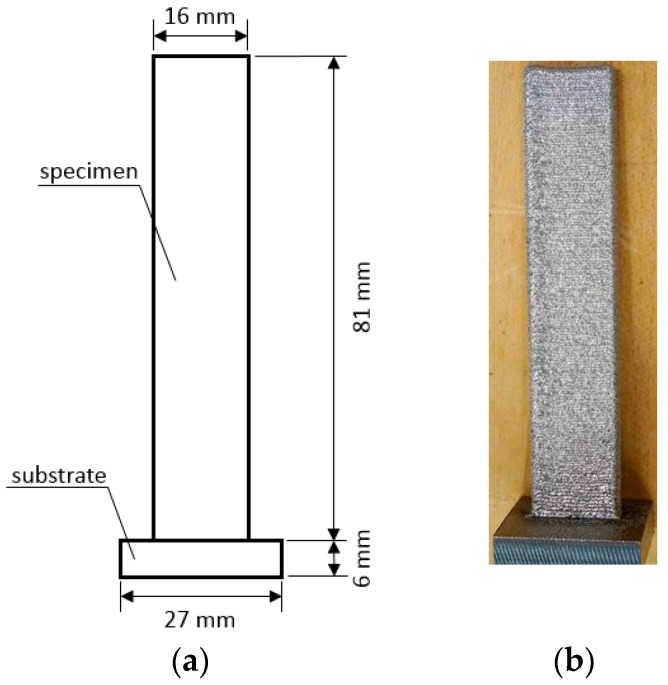
(**a**) Dimensional drawing of the as fabricated LENS specimen; (**b**) picture of LENS sample deposited on the substrate.

LENS samples were fabricated vertically-upward with a 4 × 16 mm cross-section and a length of 81 mm. Figure 1 shows the raw specimen deposited over a 6 mm-thick substrate, which was then removed by machining. LENS process can easily induce internal residual stresses within the parts during the building operations due to significantly high temperature gradients and cooling rates. Therefore, all LENS Ti-6Al-4V specimens were submitted to a stress-relief annealing heat treatment for one hour into a preheated furnace at 600 °C (~1100 °F) with a following cooling at room temperature [14].

**Figure 2:**
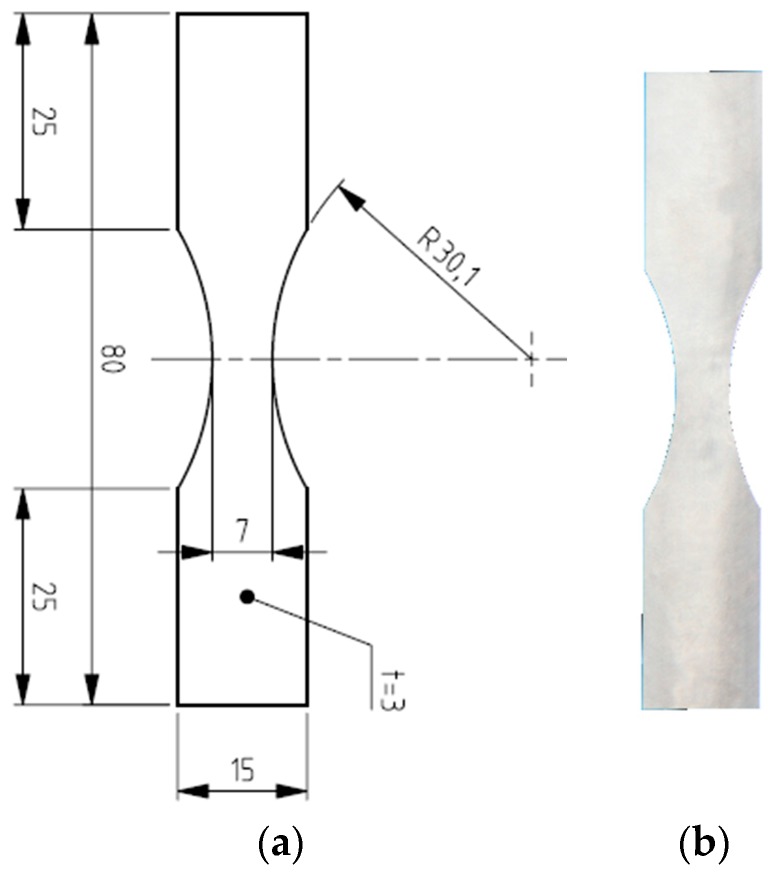
(**a**) Dimensional drawing of the specimen after post-machining; (**b**) picture of the LENS specimen after machining.

After the heat treatment process, all LENS Ti-6Al-4V specimens were post-machined via milling obtaining dimension and configuration as in Figure 2 (ASTM E 606-92 standard). The mean roughness on the specimens’ surface was measured to be 0.25 μm in order to minimize the effects of this parameter on the fatigue life. The third batch of Ti-6Al-4V specimens was built with conventional milling processes to compare fatigue performance, mechanical properties, and morphological characteristics of the fracture surface. The wrought samples had a mean surface roughness similar to that of the AM samples. 

### 2.2 Tensile and Fatigue Testing Setup

Tensile tests were conducted with a strain rate of 0.0001 s^−1^ using an Instron 5969 universal testing machine (Norwood, MA, USA) with a 50 kN load cell. Strain measurements were performed according to ASTM E8/E8M standard. For each case, three samples were tested under static load. Fatigue tests were also conducted using the same testing configuration. All tests were conducted under load control at a frequency of 10 Hz with a stress ratio R = 0.01. For each case, twelve samples were tested to obtain the S-N curve. 

## 3. Results and Discussion

### 3.1. Tensile Tests Results

The measured mechanical properties under uniaxial tension for all samples are given in Table 2. According to the static test results, porosities affected the overall mechanical behavior of the additively manufactured samples by reducing the ductility of the material. It is worth mentioning that the elongation at failure of the porous samples was almost one third of the value observed for the AM non-porous sample.

### 3.2. Fatigue Tests Results

**Figure 3:**
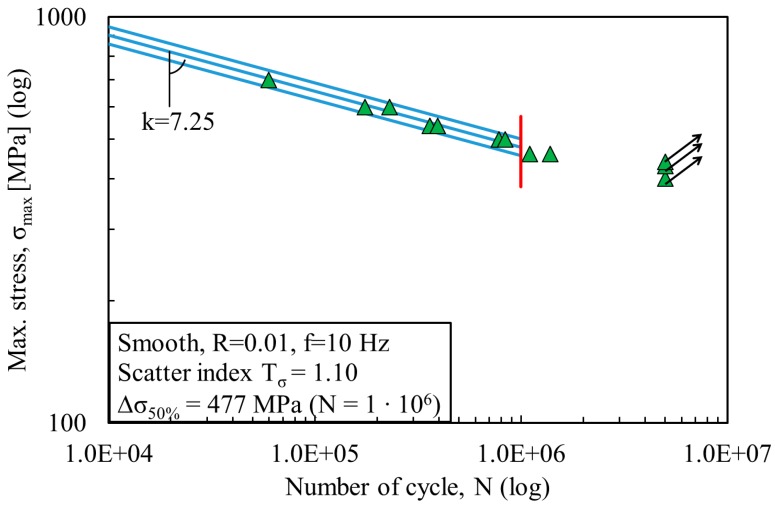
S-N fatigue data from LENS^®^ additively manufactured non-porous specimens.

**Figure 4:**
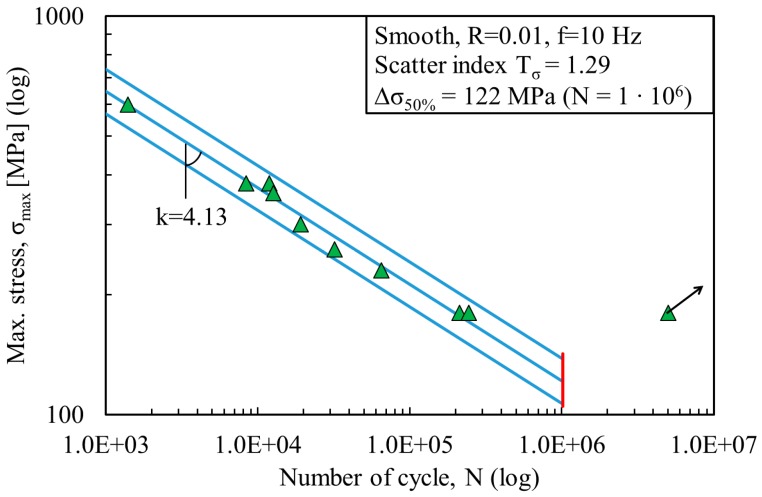
S-N fatigue data from LENS^®^ additively manufactured porous specimens.

**Figure 5:**
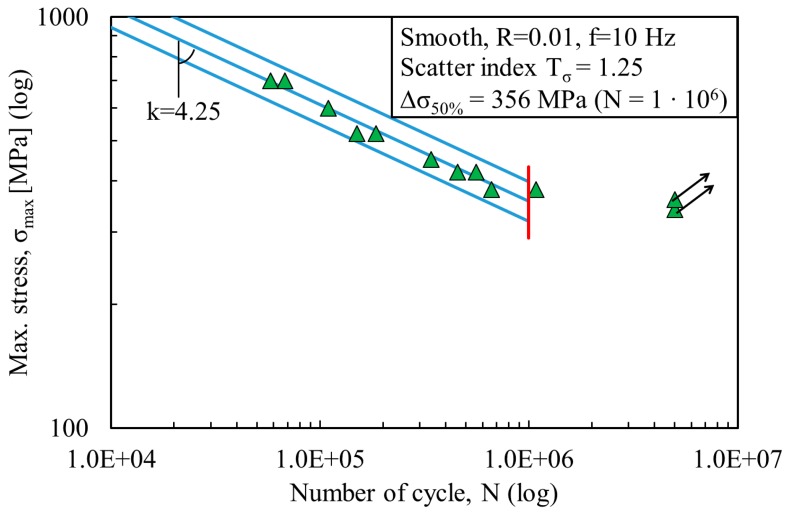
S-N fatigue data from wrought specimens.

The fatigue data were statistically investigated using a log-normal distribution and are plotted here in a double log scale. All stress ranges refer to the net area. Figure 3 and Figure 4 show the fatigue data of the non-porous and porous specimens fabricated by LENS. Figure 5 shows the fatigue data of the wrought Ti-6Al-4V specimens. The same figures depict the mean Wöhler curve (probability of survival P_s_ = 50%) and the Haibach scatter band referred to 10% and 90% probability of survival (for confidence level equal to 95%), respectively. The run-out samples, over 1 million cycles, were not included in the statistical analysis and are marked with a horizontal arrow. A vertical line indicates the values corresponding to 1 million cycles. Values of inverse slope k and scatter index T_σ_ are also shown in Figure 3, Figure 4 and Figure 5. Table 3 presents the detailed fatigue properties of the tested samples.

By comparing the results from LENS non-porous and porous specimens, a reduction of about 75% of the mean value of the stress range at 1 million cycles is observed. It decreases from 477 MPa (for non-porous specimens) to 122 MPa (for porous specimens). The inverse slope of the two curves is also significantly different, k = 7.25 for the non-porous specimens and k = 4.13 for the porous specimen. In both cases, the scatter index is limited, with T_σ_ = 1.10 for non-porous specimens and T_σ_ = 1.29 for porous specimens. The fatigue strength and life limit are about 4 times less, and this effect can be attributed to the presence of porosity. From the high values for inverse slope k and stress Δσ_50%_, it is evident that LENS non-porous specimens demonstrate a significantly different fatigue behavior than wrought specimens with much higher performance. The inverse slope k and stress Δσ_50%_ for the wrought samples are k = 4.25 and Δσ_50%_ = 356 MPa, respectively (Figure 5). LENS porous specimens also show a similar value for the inverse slope k (k = 4.13), but yet a reduction in fatigue strength (Δσ_50%_ = 122 MPa). Hence, porosity is the main contributor for this significant reduction of fatigue life. 

### 3.3. Fracture Surface Analysis and Microstructure

**Figure 6:**
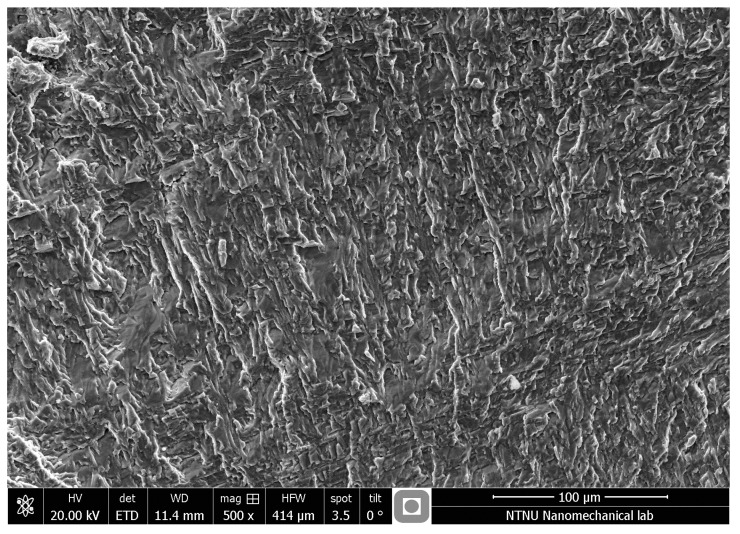
Fracture surface from a LENS non-porous flat specimen under uniaxial loading.

**Figure 7:**
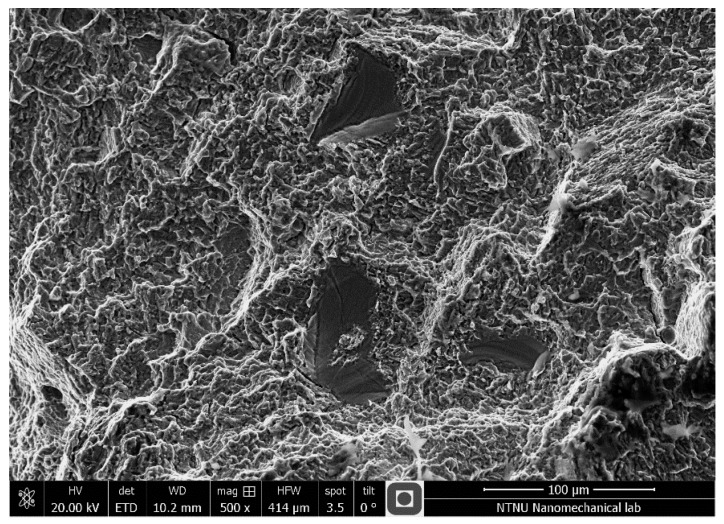
Fracture surface from a LENS porous flat specimen under uniaxial loading.

The fracture surfaces of the specimens were examined after each fatigue test. Figure 6 and Figure 7 show the typical fracture surface occurring in non-porous and porous specimens under uniaxial tensile loading. The pictures were taken from a region close to the surface of the samples where the fatigue cracks initiated. Unlike the non-porous samples, which represented a uniform fracture surface (see Figure 6), the AM porous samples had a rough surface covered by deeper dimples compared to the non-porous samples. Presence of dimples in both samples reveal the ductile behavior of the material. The presence of voids and lack of proper penetration during the manufacturing process prevented complete fusion of the particles between the layers. The presence of voids, which are local stress concentrations, facilitated the propagation of cracks in AM porous samples. It should be mentioned that the same failure mechanisms were observed for wrought samples and non-porous samples.

As a future work, additional experiments could be conducted on porous samples made by different AM techniques to conclude which approach can better control the porosity and to compare the sensitivity of materials to the induced porosity in different manufacturing techniques.

## 4. Conclusions

The fatigue behavior of Ti-6Al-4V samples produced by LENS was analyzed. Two series of AM samples, one with induced porosity and one non-porous, were fabricated using different fabrication parameters. The test results of AM samples were assessed and compared with the wrought samples. According to the static test results, the non-porous samples had mechanical properties comparable to wrought specimens. However, the porous samples had significantly lower ductility of around one third of the non-porous samples, a result of stress concentration in the material. Although the static strength of porous samples and non-porous samples were almost identical, considerably lower fatigue strength was observed for porous samples. The fatigue strengths of non-porous and porous samples were 477 MPa and 122 MPa, respectively. The induced defects in the porous samples act as stress raisers facilitating fatigue crack initiation and fatigue crack propagation along the net section of the sample. SEM has been used to investigate the fracture surface of failed samples to identify the fracture mechanisms. As a result of presence of unmolten particles and lack of penetration between the sintered layers, the AM porous samples had a rough surface covered by deeper dimples compared to the non-porous samples. However, a ductile failure was observed in both cases.

## Figures and Tables

**Table 1:** LENS processing parameters.

Fabrication Parameter	AM Non-Porous Specimens	AM Porous Specimens
Laser power (W)	325	250
Head write speed (in/min)	25	40
Layer height (in)	0.02	0.02
Hatch spacing (in)	0.015	0.03
Powder flow rate (g/min)	1.9	5.9

**Table 2:** Mechanical properties of all Ti-6Al-4V specimens.

Series	Ultimate Tensile Strength (MPa)	Yield Stress (MPa)	Elongation to Fracture (%)
LENS non-porous	1068 ± 84	914 ± 66	6.52 ± 0.31
LENS porous	987 ± 97	960 ± 94	1.95 ± 0.28
Wrought	1089 ± 92	1062 ± 83	7.46 ± 0.43

**Table 3:** Results from fatigue tests at 1 × 10^6^ cycles.

Series	N	k	T_σ_	Δσ_50%_
Wrought	2 × 10^6^	4.25	1.25	356
LENS non-porous	2 × 10^6^	7.25	1.10	477
LENS porous	2 × 10^6^	4.13	1.29	122
